# Modeling Adolescent Development and Alcohol Use in Animals

**Published:** 2000

**Authors:** Linda Spear

**Affiliations:** Linda Spear, Ph.D., is a Distinguished Professor and chair of the Department of Psychology and a member of the Center for Developmental Psychobiology at Binghamton University, Binghamton, New York

**Keywords:** animal model, adolescent, AOD (alcohol or other drug) use, psychological development, neural development, sensation-seeking behavior, risk-taking behavior, frontal cortex, stress, neurobehavioral theory of AODU (AOD use, abuse, and dependence)

## Abstract

Though certain characteristics found in human adolescents are clearly unique, there are other key characteristics of this developmental stage that are common across a number of species. Animal models offer researchers unique insight into the effects of alcohol on the adolescent. This age period is particularly important for study, because this is the time during which many people first experiment with alcohol. It is possible that features of the adolescent brain may in fact predispose a youngster to behave in ways that place him or her at particular risk for experimenting with alcohol or other drugs. In addition to behavioral changes, a number of important physiological alterations occur during adolescence, including changes in brain regions implicated in modulating the reinforcing effects of alcohol and other drugs of abuse.

The transition between childhood and adulthood is associated with a variety of developmental challenges. During this time, children—as well as youngsters from a variety of species—acquire the behavioral skills necessary to enable them to live independently, away from parental caregivers. Though certain characteristics found in human adolescents are clearly unique, there are other key characteristics of this developmental stage that are common across a number of species.

Animal models offer researchers unique insight into the effects of alcohol on the adolescent. Adolescence is particularly important for study because it is during this time that many people first experiment with alcohol. It is possible that features of the adolescent brain may in fact predispose a youngster to behave in ways that place him or her at particular risk for trying alcohol or other drugs. These characteristics may have evolved to enable the adolescent to conquer this critical developmental transition. For instance, like their human counterparts, rats undergoing the developmental transition of adolescence show a marked increase in the amount of time spent in social investigation and interaction with peers. They also are more likely to engage in risk-taking behavior, and they seek out new situations and explore unknown areas more avidly than they would at either a younger age or in adulthood.

In addition to these behavioral changes, a number of important physiological changes occur during adolescence, including significant hormonal and neural alterations. Brain areas that show particularly marked alterations during adolescence are the prefrontal cortex (PFC) and mesolimbic regions of the forebrain in which dopamine (DA), a key brain chemical, is found. These regions also have been implicated in modulating the reinforcing effects of alcohol and other drugs of abuse.

This article begins by defining the period known as adolescence and drawing distinctions between adolescence in humans and in other animal species. Next, the article reviews research findings from studies of animal models of alcohol and adolescence and discusses the need for developing adolescent models of alcohol effects in other species as well.

## Defining Adolescence

In general, adolescence can be defined as the gradual period of transition between youth/immaturity and adulthood. There is a tendency to associate adolescence with puberty. Yet the process of adolescence is not synonymous with puberty. Instead, puberty is but one of several important developmental changes that occur along the timeline that comprises adolescence. In fact, many of the behaviors that define adolescence, such as risk taking, alcohol and drug use, and considerable peer influence, are common in settings such as college and the military as individuals move toward autonomy, and these individuals are well past sexual maturity. Thus, whereas some clinical researchers define adolescence in humans as the age span from approximately 9 to 18 years of age (see e.g., Buchanan et al. 1992), others consider the entire second decade of life as “adolescence” (e.g., Petersen et al. 1996). Even ages up to 25 years have been considered as “late adolescence” by some researchers (Baumrind 1987).

The exact timing of adolescence is a matter of some dispute in laboratory animals as well (Odell 1990). Spear and Brake (1983) defined periadolescence as the age around the time of sexual maturation when age-specific behavioral and physiological changes are evident. According to these criteria, periadolescence in rats was defined as approximately 30–42 postnatal days (i.e., P30–42), although animals, like their human counterparts, may show some signs of adolescence at significantly younger and older ages, with male rats tending to mature more slowly than females. Indeed, age-typical alterations characteristic of adolescence may begin as early as P28 and may last in male rats until P55 or so (see, for example, Ojeda and Urbanski 1994). In the monkey, adolescence is recognized as occurring in the age range of 2–4 years (Lewis 1997).

Obviously, one of the major challenges in modeling adolescence lies in defining this age group. It simply may not be possible, however, to develop a model that exactly matches all of the behavioral and physiological changes taking place in human adolescents. Instead, the animal model chosen and its developmental timeline may be dependent on the particular adolescent-related characteristic that is being modeled. Examples of typical adolescent features seen in humans that may be modeled in other animal species are outlined in the sections below.

## Behaving Like an Adolescent

Certain behaviors clearly are unique to adolescents, whether they pertain to rats, monkeys, or humans. Human adolescents spend substantially more time interacting socially with peers than with adults. Such peer-directed social interactions may help the adolescent develop social skills away from the home environment and hence ease the transition toward independence (Larson and Richards 1994). Social interactions, particularly with peers, increase in importance during adolescence in many species. For example, rats in the age range of about P30 to P42 spend considerably more time in social interactions (Primus and Kellogg 1989) and at play (e.g., Fassino and Campbell 1981). Those interactions give the adolescent rat the opportunity to practice and model adult-typical behavior patterns (Galef 1981). Sex differences in behavior also typically begin to emerge during this time, presumably at least in part as a result of increased hormonal activity during puberty.

## Adolescence Is Risky Business

As they grow, adolescents across a variety of species increasingly seek out new situations and sensations (i.e., novelty/ sensation seeking) and risks (Arnett 1992; Spear 2000). As a function of evolution, these tendencies may provide the impetus to explore new and distant areas, helping to avoid inbreeding by ensuring the dispersal of male (and sometimes female) offspring to territories away from animals with whom they are closely genetically related. Increased risk taking also gives the adolescent the opportunity to explore new behaviors, which may help him or her relinquish childhood patterns of behavior in favor of behaviors essential for functioning as an adult. The kinds of risks that human adolescents take not only include behaving recklessly, acting up in school, and behaving antisocially (e.g., fighting, stealing, trespassing, and damaging property) but also using alcohol or other drugs. Shedler and Block (1990) have proposed that modest amounts of risk taking may represent “developmentally appropriate experimentation.” According to the researchers’ findings, adolescents who engaged in moderate amounts of risk taking were found to be more socially competent both in childhood and adolescence than were abstainers or more frequent risk takers. Thus, whereas risk-taking behavior may have a constructive role— at least in an evolutionary sense and, arguably, also within human adolescence—excess risk-taking may be disadvantageous, if not life threatening, both for the adolescent and for other people affected by the adolescent’s behavior.

## Initiating Alcohol or Other Drug Use

As with other types of risk-taking behavior, some amount of exploratory alcohol or other drug use is common in human adolescents. In fact, alcohol use and abuse, as well as alcohol-related problems, are highly prevalent among American youth (O’Malley et al. 1998). According to O’Malley and colleagues, the prevalence of alcohol use and of having been drunk increases sharply during adolescence. A considerable proportion of 8th graders already uses alcohol, with one-fourth of the students reporting having had a drink in the past 30 days. Moreover, one in three of those drinkers reported having consumed enough alcohol to get drunk or very high. More than one-half of the 12th graders reported having had at least one drink—and more than one-third reported having been drunk at least once—in the past 30 days (for a review, see O’Malley et al. 1998).

This early alcohol use may have potentially long-lasting consequences. Early onset of alcohol or other drug use is one of the strongest predictors of later alcohol dependence (Grant 1998). In other words, the earlier a person begins drinking alcohol, the more likely he or she is to become dependent on alcohol. Moreover, this early onset of drinking was linked to an increased risk of dependence regardless of a family history of alcoholism; that is, people who drink at an early age are not necessarily destined to become alcohol dependent simply because they have family members who are alcohol dependent.

There are at least two possible explanations for this powerful early exposure effect. First, exposure to alcohol or other drugs during adolescence may alter critical ongoing processes of brain development that occur at that time, increasing the likelihood of problems with alcohol later in life. Indeed, heavy drinking during early and mid-adolescence has recently been found to be associated with memory problems and other neuropsychological deficits, although the causality of this relationship has yet to be determined (Brown et al. 2000). Indirect support for the notion that early alcohol use may itself increase later alcohol problems comes from analysis of data from 10- to 11-year-old children collected prospectively for 7 years. In that study, the influence of all significant risk factors for alcohol misuse were found to be linked to the age at which alcohol use was first initiated, the only exception being a modest, independent influence of gender (with males misusing alcohol more than females) (Hawkins et al. 1997).

An alternate interpretation for the early exposure effect is that early use of alcohol or other drugs might simply serve as a marker, not a precursor, for a later abuse disorder. For instance, high novelty-seeking behavior in preteens was predictive of alcohol abuse at age 27 (Cloninger et al. 1988); high novelty seeking is one of a number of traits that are linked to initiation of alcohol and other drug use (Baumrind 1987).

These two views on the significance of the early exposure effect are not necessarily mutually exclusive. For instance, the presence of behavioral problems (i.e., conduct disorder problems) is associated with an increased probability both for the early use of alcohol and other drugs as well as for later alcohol and drug abuse. Yet, among people with conduct disorder, those who begin using alcohol and other drugs at an early age have an especially high risk for problems with alcohol and other drugs later in life. (Robins and McEvoy 1990).

In animal studies, genetic differences in alcohol intake among inbred lines of rats have been observed not only in adulthood but also in adolescence (McKinzie et al. 1999), raising the possibility that similar genetic factors might influence not only problem drinking in adulthood, but also the emergence of alcohol drinking during development. Indeed, Prescott and Kendler (1999) recently concluded from studying human twins that the age of initiation of alcohol use was not a direct risk factor for alcoholism, but was an “alternative manifestation of vulnerability to problematic alcohol involvement” (p. 106). According to this view, to arrive at a conclusion that early drinking causes later alcohol dependence, the age that the person first begins drinking must be tied only to that person’s own idiosyncratic (i.e., individual-specific) characteristics apart from any genetic or environmental influences that would be shared with the other twin. Given this assumption, the researchers’ concluded that the association between drinking onset and later alcohol dependence reflected not just individual-specific variation but also the shared genetic and environmental factors that existed between the twins. Thus, unlike the Grant study mentioned earlier, Prescott and Kendler concluded that there was no causal relationship between drinking onset and alcohol dependence.

Animal models could help determine whether or not there is indeed a causal relationship between early exposure and later alcohol problems and could help reveal the mechanisms underlying this possible association. Although not well investigated, some evidence exists that drinking during adolescence has a long-term influence on later neurobehavioral function. For instance, voluntary alcohol consumption (Shtiegman et al. 1997) during adolescence was found to increase later aggressive behavior significantly in male Golden hamsters. Chronic alcohol exposure in adolescent rats was reported to induce long-lasting alterations in cognitive functioning (Osborne and Butler 1983) and disrupt puberty-associated increases in reproductive endocrinology in both males (Cicero et al. 1990) and females (Dees et al. 1990). In most cases, it is not clear, however, whether exposure during adolescence is critical for these effects or whether similar effects would be evident with comparable alcohol exposure in adulthood.

Of particular relevance for understanding the early exposure effect is the little explored question of whether adolescent alcohol exposure alters later alcohol sensitivity or intake. Reports in rodents that pre- and post-weaning (Hayashi and Tadokoro 1985; Ho et al. 1989) alcohol exposure can increase the animal’s preference for alcohol later in life are contrary to other data showing no increases in later consumption after periods of alcohol exposure at times that include adolescence (Tolliver and Samson 1991). In the development of animal models, it may prove useful to consider the intriguing suggestion made by Tolliver and Samson (1991) that stress may have an effect on early exposure and on later patterns of alcohol or other drug use. The following sections address the important role that stress plays in adolescence and in the development of alcohol and other drug problems.

## Stress and Adolescence

Navigating the often-stormy transition from the dependence of childhood to the independence of adulthood can be a stressful experience for adolescents. In humans, for example, levels of anxiety have been reported to peak at around 13 to 15 years of age (see Buchanan et al. 1992 for discussion and references). Incidence of depressed mood also increases notably from childhood to adolescence to reach rates that are often higher than in adulthood (Petersen et al. 1993). Even physiologically, adolescents may show an increased response to stressful situations. For example, human adolescents exhibited higher increases in blood pressure and cardiac output—key indicators of stress—in response to a variety of stress-simulated laboratory tests than did younger children (Allen and Matthews 1997).

Researchers have postulated that the presumed increase in anxiety and stress during adolescence contributes to the frequent initiation of alcohol or other drug use observed in adolescents (see, for example, Pohorecky 1991; Wagner 1993), as well as to the frequent emergence in adolescence of schizophrenic symptomology in people at risk for psychiatric disorders (Walker and Diforio 1997). In her review of the literature on stress effects on alcohol consumption in humans, Pohorecky (1991) concluded that stress is most convincingly associated with alcohol consumption in adolescence, with more mixed findings evident in studies conducted in adults. Indeed, after peer substance use, the most powerful predictor of adolescent alcohol and drug use, as found by Wagner (1993), was levels of perceived stress. In addition, Wagner found that the adolescent’s perception of the event as being stressful was of more importance than the absolute number of such events.

Although limited in number, studies in laboratory animals also show that adolescents are more negatively affected by stressful events than are adults. For instance, compared with adults, adolescent rats show more immobility under stressful situations, such as a forced swim test (Walker et al. 1995). Stone and Quartermain (1998) reported that chronic social stress (brought on by a short daily encounter with a strange adult male mouse for 5 days) had a greater impact on adolescent (i.e., P28–32) than on adult male mice. The higher stress levels resulted in greater reductions in food intake, body weight gain, and time spent on the open arms of a plus maze—an indicator of anxiety—in adolescents than adults. Likewise, studies in laboratory animals have shown that adolescents sometimes exhibit a greater overall hormonal response to stress—evidenced by the increased production of a key stress-related hormone, corticosterone—compared with younger animals and a more prolonged increase in stress hormones relative to adults (Spear 2000).

In addition to stressful events, pleasurable experiences, such as drinking alcohol, also increase stress hormone levels in laboratory animals (e.g., Ellis 1966) and humans (e.g., Schuckit et al. 1987). In fact, corticosterone itself has been shown to be reinforcing and is self-administered by rodents both intravenously and orally (Deroche et al. 1993; Piazza et al. 1993). Indeed, Piazza and Le Moal (1997) view corticosterone as a naturally occurring psychostimulant with neurochemical and physiological effects similar to certain drugs such as cocaine and amphetamine.

It may be highly significant that several studies have incidentally noted that adolescent rats exhibit an attenuated corticosterone response to alcohol and other drugs than do their more mature counterparts (Bailey and Kitchen 1987; Silveri and Spear 1999). Considering that increases in corticosterone contribute to the rewarding aspects of alcohol, adolescents might need to consume more alcohol to achieve the same effect that lower levels would have in more mature individuals. This notion implies however, that the rewarding effects of alcohol and other reinforcing drugs would progressively increase with increasing elevations in cortiocosterone levels, which may not be the case (see Goeders and Guerin 1996). It remains to be seen whether the reduced corticosterone response to alcohol seen in adolescent laboratory animals is also evident in humans and might contribute to the higher-per-occasion levels of alcohol use (i.e., binge drinking) found in human adolescents.

Like the body’s hormonal systems, many of the neural systems known to undergo developmental changes during adolescence are also activated by stress, including dopamine (i.e., DA) projections to prefrontal cortex or PFC (for further information, see the next section, “Adolescent Brain—Unlike Any Other”), as well as to mesolimbic brain regions (Abercrombie et al. 1989)—areas thought to be critical in modulating the pleasurable response that follows alcohol use (Koob 1992). Important docking molecules (i.e., receptors) for the stress hormone corticosterone have been identified in rodent brains on DA cell bodies in the ventral tegmental area and substantia nigra as well as in DA terminal regions, including the nucleus accumbens and the PFC (Ahima and Harlan 1990; Cintra et al. 1994). Increases in corticosterone may play a critical role in activating DA transmission, as evidenced by the fact that treatment with corticosterone increases and removal of the adrenals (the area where corticosterone is produced) decreases DA levels in the nucleus accumbens (Piazza et al. 1996*b*) and PFC (Imperato et al. 1989) of rodents. Likewise, adrenalectomy or pharmacologically induced blockade of stress-hormone synthesis suppresses alcohol consumption (Fahlke et al. 1994) in laboratory animals. The results of this basic research suggest that stress-induced increases in the stress hormones may interact with mesocorticolimbic brain regions to facilitate alcohol-use behavior. Further research into the effects of stress on the development of alcohol problems is crucial. Investigations of stress effects in adolescents will be especially important given the dramatic changes taking place in the brain during that time, as discussed in the following section.

## Adolescent Brain—Unlike Any Other

As children make the transition to adulthood, the outward signs of growth are clearly visible. Yet even more dramatic than the often striking physical changes occurring in adolescents are the changes taking place in their brains. This remodeling of the brain during adolescence appears to be similar across species and includes a growth or maturing of some brain constituents (such as the formation of additional connections between nerve cells) as well as a prominent loss (or pruning) of some existing connections.

The adolescent-associated changes in DA input to PFC and limbic brain regions (i.e., the so-called mesocorticolimbic DA system) may have a profound effect on adolescent behavior and psychological functioning. In rats, this stress-sensitive DA system has been implicated in novelty seeking (Dellu et al. 1997) and as part of a brain cell circuitry that is involved in assigning value (i.e., “incentive salience”) to stimuli, including alcohol, and translating this decision into action (Kalivas et al. 1993).

A key brain area that is prominently remodeled during adolescence is the prefrontal cortex (PFC), a region thought to be involved in various goal-directed behaviors (e.g., rule learning, working memory, and spatial learning) and in emotional processing, particularly of unpleasant stimuli. Along with a decline in the relative size of portions of the PFC during adolescence, there is substantial remodeling of communication connections between nerve cells in this region—with some childhood connections lost and others added. For example, as demonstrated in nonhuman primates, the input from two key chemicals (i.e., neurotransmitters) involved in brain cell communication—the excitatory neurotransmitter glutamate and the inhibitory neurotransmitter gamma-aminobutyric acid (GABA)—is reduced during adolescence, whereas the input from another neurotransmitter, DA, peaks in PFC during adolescence (Lewis 1997). Developmental adjustments also are evident in limbic brain regions and occur across a variety of species. One of those regions includes the amygdala (Yurgelun-Todd 1998), a complex grouping of brain cells that among other things, is thought to be involved in a person’s emotional reactions and in coordinating the body’s response to stress.

Given that adolescence is associated with important alterations in the PFC, limbic brain areas, and the DA input to these regions, concomitant alterations in motivated behaviors also might be expected. Alterations in the incentive value assigned to stimuli could underlie many of the behavioral alterations seen in adolescents. For example, those alterations may lead to the increased importance that adolescents assign to peer relationships and to their increased need to seek out new and often risky experiences, such as initiating alcohol or other drug use. Because the functioning of these brain areas is so different between adolescents and adults, it would be astonishing indeed if adolescents did not differ from adults in various aspects of their motivated behavior.

Currently many gaps exist in our knowledge of the developmental changes that occur in the brain during adolescence. Still, the data that are available suggest that the prominent alterations which occur during adolescence in brain regions such as the PFC take place not only in humans but also in other species as well, ranging from rodents to non-human primates. Given these across-species similarities in neurobehavioral features of adolescence, the question arises whether nonhuman animals undergoing this developmental transition can be used as models of human adolescence. The following sections summarize findings from animal models of human adolescence, including the gaps that exist in current research.

## Animal Models of Alcohol’s Effects on Adolescent Development

Some researchers have argued that adolescence is uniquely human and hence cannot be modeled in animals (e.g., Bogin 1994). For example, Bogin maintains that only humans undergo adolescence based on the conclusion that only human adolescents show a growth spurt (von Bertalanffy 1960). Yet, common markers of such a growth spurt, such as developmental overeating (i.e., hyperphagia) and accelerated growth rates, are found even in adolescent rodents (e.g., Kennedy 1967). Other investigators have concluded that pubertal growth spurts are common across mammalian species, but argue that it is the relatively long period of slowed growth during the preadolescent childhood/early juvenile period that is unique to humans and other primates (Weisfeld and Billings 1988). Before basing the appropriateness of animal models for the study of adolescence on growth criteria alone, researchers also must consider whether the global attributes of adolescence should be affirmed or dismissed based solely on a single characteristic.

For example, a myriad of important processes unfold during adolescence, and what one person identifies as essential features(s) of adolescence will determine the appropriateness of any given animal model. Traditionally, animal models have been used extensively for modeling human psychopathology, with each animal model typically emulating only a handful of psychopathological features thought to be central to the target disorder. Certainly, no animal model can be similar in all respects to the complexity of human psychopathology or to human behavior during adolescence (or at any other time of life). Indeed, certain areas of human adolescent functioning may never be addressed using animal models, including the role of peer pressure and self-esteem, the impact of parenting styles, the obsession with thinness found in adolescent females in some Western cultures, and cultural differences in how adolescents are perceived, to mention a few.

Thus, as with animal models of psychopathology in humans, before researchers can determine whether a particular animal model will be useful, it is first necessary to consider what aspect of human adolescence is to be modeled. For example, considering that much information about the brain chemicals and hormonal factors that modulate alcohol and other drug administration have been obtained from rodent studies, it is likely that rodents will provide a useful and cost-effective tool for examining alcohol use in adolescents. Such research is just beginning. Using the adolescent rat as a model, adolescents have been shown to accommodate more rapidly to the presence of alcohol in their system (so-called “acute tolerance”) than adult rats do, thereby reducing adolescent relative sensitivity to the motor impairing and sedative consequences of alcohol (Silveri and Spear 1998). Yet, whereas this insensitivity to alcohol impairment may permit adolescents to drink relatively large amounts when compared with their more mature counterparts, research with rodents has shown that this exposure has more adverse effects on hippocampally related memory processes in adolescents than in adults (Markwiese et al. 1998; Swartzwelder et al. 1995). Research using rodents could examine not only mechanisms underlying age differences in sensitivity to alcohol’s impairing and reinforcing effects, but also the influence of social interactions, changes in the environment, and the impact of stressful events on alcohol use during adolescence.

On the other hand, key brain systems of rodents are substantially less prominent, their social organization is considerably less complicated, and the time course of their adolescence is far briefer than those of humans or of nonhuman primates. These and other limitations put constraints on the use of rodent models. The relatively longer developmental period of nonhuman primates and their generally more complex social structure relative to other laboratory animals may make this species more useful for long-term studies of pharmacological and social environmental influences on alcohol self-administration in adolescents. More must be understood regarding the course of adolescence in nonhuman primates, however, particularly among seasonal breeders where the onset of puberty (i.e., reproductive maturity) is often tightly synchronized with the mating season (e.g., Plant 1996).

The validity of animal models is typically assessed using three evaluation criteria borrowed from the psychological-testing literature (Wiggins 1973) and adapted for assessing the validity of animal models in human psychopathology (e.g., Willner 1991):

- *Face validity*, which asks whether there are similarities between the model and what is being modeled in terms of etiology, symptomology, treatment, or physiological bases
- *Predictive validity*, which examines whether the model successfully forecasts future findings (typically regarding the efficacy of drug treatments)
- *Construct validity*, which addresses whether the model is homologous to the clinical syndrome being modeled in terms of physiological determinants, precipitating psychosocial environment, and other factors thought to influence its occurrence.

There are many similarities between human adolescents and a number of animal models of adolescents in terms of developmental history, behavioral symptomology, and neural and hormonal characteristics. Those resemblances provide some measure of face and construct validity that are sufficiently promising to support further development of these animal models as tools for the study of adolescence. Assessment of validity is an ongoing process; as more data are generated, stronger tests of construct validity as well as of predictive validity will be possible. Ultimately the validity of animal models is determined by their usefulness in expanding the understanding of the phenomena under investigation, propagating further testable hypotheses, and generating data to refute or refine the model and further assess its validity.

## Summary and Conclusions

Research on alcohol’s effects on the developing adolescent is still in its infancy, despite the fact that this is the time during which many youngsters begin drinking. There is evidence that people who begin drinking at an early age may have problems with alcohol later in life. Research also has shown that adolescence is a time during which remarkable changes are taking place in the brain. Just how alcohol use impacts this development is unknown. Clearly additional research is needed, and animal models offer an ideal means for gathering information on alcohol’s effects on development. The following paragraphs outline specific areas in need of additional investigation, in both animal models and in human subjects.

To date, research conducted largely in laboratory animals has shown that a number of striking alterations occurs in the brain during the time period that is representative of adolescence in humans. Many of these observations have been made piecemeal. The existing findings still need to be more fully characterized and then integrated so that a complete picture of the changes occurring in the brain can be developed. It also remains to be determined how these changes impact behavior. For example, what are the functional implications of alterations in mesocorticolimbic regions during adolescence? How widespread are adolescent-associated changes among other systems and brain regions? Evidence exists to suggest that alterations during adolescence in forebrain regions (such as the PFC and mesocorticolimbic DA systems) play a role in alcohol’s rewarding (or pleasurable) effect. Still, little is known about how this may precipitate and maintain alcohol use during adolescence.

Studies using animals also have shown that compared with other age groups, adolescents do not experience the same degree of incoordination and sleepiness when drinking alcohol as do adults (that is, adolescents are relatively resistent to the motor impairing and sedative effects of alcohol). They do, however, appear to be more sensitive to alcohol-induced disruptions in spatial memory. Further research is needed to determine when youngsters in this age group are most susceptible to alcohol’s effects as well as the mechanisms that underlie their responses to alcohol. This work also should examine how alcohol tolerance and sensitization develop during adolescence and the impact of these adaptations on the initial and subsequent responses to alcohol by adolescents. Understanding tolerance and sensitization is particularly important given that research has suggested that a lower level of intensity of reaction to alcohol may increase the likelihood that a person will drink more heavily and more often, setting the stage for the development of alcohol problems (Schuckit 1995).

Clinical evidence shows that stress is more strongly associated with alcohol consumption in adolescents than in adults, with stress being among a number of risk factors contributing to the initiation and continuation of alcohol use in this group. Yet investigations are lacking on how stressful situations may lead to drinking in youngsters. Researchers need more information about the hormonal, behavioral, and neural interactions that take place in response to stress during adolescence. Understanding why youngsters use alcohol to cope with stress within a developmental timeframe also is important. Conclusions about the relationship between stress and adult drinking may be far different from the relationship between these variables in adolescence, the time when most alcohol use is initiated.

Understanding how changes in brain function interact with a youngster’s sensitivity to alcohol’s effects and his or her response to stress is another key area for future research and one that would be ideal for animal studies. For example, what factors trigger these critical developmental changes in brain function? Do increases in puberty-associated hormones play significant roles in altering the stress response characteristics of adolescents?

It is critical that future research efforts (1) examine why early exposure to alcohol is apparently so much more dangerous than later use and (2) determine whether early exposure does indeed increase a person’s propensity for later alcohol problems. These two issues are especially relevant for the design of successful prevention efforts.

Multidisciplinary research efforts are needed to fully explore the neurobehavioral and environmental factors influencing alcohol sensitivity and alcohol use during adolescence as well as lasting consequences of this use. Animal models will be able to mimic some important aspects of adolescence. For example, rodent studies can be used to rapidly and cost-effectively characterize many of the neuronal, hormonal, and behavioral features of adolescence, as well as the interrelationships among these factors, environmental stressors, and their association with the use and later abuse of alcohol. The brevity of the adolescent period in rodents, however, presents a challenge for the design of some experiments. Thus, for some research questions, it may be preferable to turn to the use of nonhuman primates. Primates typically develop at a rate that more closely resembles human development, which might prove beneficial for long-term studies of the pharmacology of adolescent alcohol use. Little is yet known, however, about neurobehavioral function of adolescent primates under typical circumstances (Pereira and Fairbanks 1993), let alone in the presence of alcohol. Corresponding human adolescent development to that of nonhuman primates will thus require further investigation.

Most important, data from animal models can provide scientists with the information needed to educate adults and adolescents alike as to the effects of youthful drinking, providing scientifically based evidence regarding whether alcohol is indeed detrimental to the developing adolescent. Parents (and adolescents) who may condone drinking—”At least it’s not drugs”—often view alcohol as a relatively harmless rite of passage. Yet research demonstrates that adolescence is a time during which remarkable changes are taking place in the body and in the brain, with the changing brain perhaps being particularly vulnerable to alcohol’s harmful effects. Animal studies could help explain why early exposure to alcohol may be more dangerous than postponing use to adulthood. In addition, animal models may provide a biological explanation for why adolescents engage in binge drinking at higher rates than adults may and determine the possible long-term consequences of this pattern of consumption.

In all of this work, it is critical for researchers to keep in mind that adolescents cannot be treated simply as immature adults. The distinctive characteristics and proclivities of adolescents must be considered when developing appropriate animal models, approaches, and techniques to study this unique and important developmental stage.
